# Editorial: Mental health services for occupational trauma: decreasing stigma and increasing access, volume 2

**DOI:** 10.3389/frhs.2025.1663204

**Published:** 2025-07-28

**Authors:** Warren N. Ponder, Natalie Mota, Shay-Lee Bolton

**Affiliations:** ^1^ One Tribe Foundation, Euless, TX, United States; ^2^ Department of Clinical Health Psychology, University of Manitoba, Winnipeg, MB, Canada; ^3^ Departments of Psychiatry and Community Health Sciences, University of Manitoba, Winnipeg, MB, Canada

**Keywords:** first responder, occupational health, public safety, health care professionals, mental health, burnout - professional, psychology, resilience

**Editorial on the Research Topic**
Mental health services for occupational trauma: decreasing stigma and increasing access, volume 2

First responders, including ambulance personnel, frontline medical workers, physicians, law enforcement officers (LEOs), and nurses are under continued public scrutiny and unprecedented stress that has been compounded by the COVID-19 pandemic (1). The continuous stress (e.g., high demand, understaffing, training and resources), frequent exposure to trauma, and burnout among these groups can have a negative impact on their health and career trajectory (2). However, many members are extremely reticent about seeking mental health treatment to address their distress (3). Burnout and mental health difficulties can manifest differently across cultures. As such, this special issue from international scholars on occupational mental health includes representation from a range of high-risk occupations from Brazilian, Chinese, Danish, South Korean, and American samples.

In a sample of Brazilian physicians, Becker et al. found that almost 12% of healthcare professionals experienced burnout, while in China, Wen et al. showed that burnout significantly mediated the relationship between work and family conflict, and job satisfaction; but that social support moderated the impact of burnout on job satisfaction. This finding underscores the importance of a supportive relationship at work and at home in mitigating the deleterious effects of burnout. Social support is also key to other frontline healthcare professions, such as nursing. In a systematic review and meta-analysis, Chen et al. found an inverse relationship between social support and turnover intention, a measure assessing the likelihood that they would leave their jobs. These results may be useful as a guide for nurse managers, healthcare centers, and policy administrators with actionable items to help reduce turnover, by encouraging and promoting social support.

Sim et al. used a sample of South Korean nurses to examine posttraumatic growth (PTG), burnout, and posttraumatic stress disorder (PTSD) during the COVID-19 pandemic. They found that purposeful rumination, emotional expression and cognitive emotional regulation (cognitive coping while not being overburdened by negative emotions), increased PTG. Furthermore, they showed that PTG was a protective factor both against burnout and persistent PTSD symptoms. Melander et al. investigated social support and PTSD in a sample of Danish ambulance personnel and found that social support predicted higher levels of PTSD symptoms, and that informal managerial and collegial support was preferential to formal social support (e.g., debriefing/defusing, formal training for peer support or a manager). Their sample also overwhelmingly preferred seeking out a family member or close friend for support. These studies further illustrate the importance of social support and protective factors against burnout.

A sizable minority of U.S. first responders—between 17% and 28%—have prior military service (4, 5), and there may be additional institutional (e.g., sensitivity, logistic, and not fitting in) and stigma-related barriers to care that should be considered (6). Ein et al. conducted a rapid review to understand barriers and facilitators related to mental health service utilization in veterans. Some examples of primary barriers included system navigation difficulties and negative attitudes toward mental health, while facilitators included mental health literacy and social support. If this population can overcome perceptions of stigma and potential negative impacts on career trajectory, recent research has recommended a transdiagnostic approach that focuses on emotion regulation (7). To help break down barriers to care, Meyer et al. sought to address stigma, logistical barriers, and lack of therapist cultural competency through implementation of the Unified Protocol in a sample of first responders. They found significant reductions in PTSD, depression, and generalized anxiety symptoms among first responders in this uncontrolled trial with treatment delivered via telehealth (Meyer et al.).

While this special issue fills some of the gaps in the literature, much more can be done. One of the most concerning consequences of burnout and untreated mental health symptoms is an increased risk of substance misuse as a coping mechanism. This can lead to serious career repercussions for members in these occupational roles, including legal consequences. To address this, Fort Worth, Texas created the first Public Safety Employees Treatment Court (PSETC), in the United States, which gives first responders an opportunity for participation in a specialty diversion court program that, if successfully completed, could dismiss their case. The program typically takes 8-to 24-months, and the participants have to adhere to their collaborative treatment plan established at entrance into the program. In the initial study, there were reductions in suicidality, generalized anxiety, depression, emotional distress, and PTSD, while resilience increased (8).

It is also important to continue to understand risk and protective factors for burnout and mental health symptoms in these populations by conducting additional international epidemiological studies. We propose that an interdisciplinary team of scholars and data analysts should leverage international samples using the same assessments for secondary data analytic comparative studies. This taskforce could function in a similar manner to what the National Vietnam Veterans Readjustment Study achieved for Vietnam veterans in the 1980s (9). Using a nationally representative sample, findings from that study elucidated the scale of mental health problems among veterans and, in 1989, led to the first VA-established National Center for PTSD in Boston (10). Since then, the National Center has been at the forefront of the continued study of trauma in veterans and evidence-based solutions to alleviate suffering from posttraumatic stress. First responders deserve the same level of scholarly investigation. We hope this special issue contributes to a larger body of much-needed work in this area.
